# Temperature-independent polymer optical fiber evanescent wave sensor

**DOI:** 10.1038/srep11508

**Published:** 2015-06-26

**Authors:** Nianbing Zhong, Qiang Liao, Xun Zhu, Mingfu Zhao, Yun Huang, Rong Chen

**Affiliations:** 1 Chongqing University of Technology, Chongqing Key Laboratory of Modern Photoelectric Detection Technology and Instrument, Chongqing 400054, China; 2 Key Laboratory of Low-grade Energy Utilization Technologies and Systems (Chongqing University), Ministry of Education, Chongqing 400030, China

## Abstract

Although the numerous advantages of polymer optical fibers have been exploited in the fields of sensors and telecommunications, such fibers still experience a critical problem: the temperature dependency. Therefore, we explored the temperature-independent operation of a polymer fiber-optic evanescent wave sensor immersed in distilled water. We investigated variations in the surface morphology, deformation trajectory, refractive index, and weight of the fiber-sensing region with varying water temperature. We also examined the spectral transmission and transmitted light intensity of fibers subjected to a heating-cooling treatment. We observed that the light-transmission modes and sensitivity of the sensor were affected by changes in the surface morphology, diameter, and refractive index of the sensing region caused by changes in temperature. The transmitted light intensity of the sensor was maintained at a constant level after five cycles of the heating-cooling treatment, after which the fibers exhibited a smooth surface, low refractive index, and large fiber diameter. Consequently, we utilized the heating-cooling-treated fiber to realize a temperature-independent, U-shaped polymer fiber-optic evanescent wave sensor. The temperature independence was evaluated using glucose solutions in the range of 10 to 70 °C. The fabricated sensor showed significant temperature independence and high degree of consistency in measuring solutions.

Fiber-optic evanescent wave (FOEW) sensors, which are composed of silica and polymer optical fibers^1^,^2^, are widely applied in chemistry^3^, biochemistry^4^, life sciences^5^, and environmental research^6^ because they provide fast and reliable results in terms of measuring solution concentrations, chemical component analysis, and obtaining the absorption spectra of chemicals of interest during real-time monitoring^7^. The sensor measurements are not affected by the bulk solution because the penetration depth of the evanescent field ranges from ten to several hundred nanometers^8^. Regarding currently available FOEW sensors, sensors based on polymer optical fibers (POFs) have attracted intense interest because of their various potential advantages, including low cost, large-diameter core, large numerical aperture, and high degree of flexibility (U-, D-, taper-, and spiral-shaped fibers can be fabricated)^9^,^10^,^11^. The large diameter and numerical aperture of the fibers can increase the optical transmission capacity of the fiber and the performance of such sensors, i.e., in terms of improved sensitivity and measurement range^12^,^13^. Although the POFs represent a promising technology in the fields of sensors and telecommunications, such fibers still face a critical problem of the temperature dependency^14^,^15^.

Various techniques have been developed to enhance the stability of POFs in high-temperature environments. Nakao *et al.*^16^ proposed a new core material consisting of P(TCEMA-*co*-*c*HMI) and diphenyl sulfide (DPS) as the dopant, and the copolymerization of *c*HMI with DPS drastically improved the thermal stability and decreased the light-transmission losses. Ishigure *et al.*^17^ reported that the use of fiber cores of POFs doped with hydrophobic materials resulted in decreased absorption of water molecules and maintenance of a low level of light attenuation under high-temperature and high-humidity conditions. Makino *et al.*^18^ also reported that dopant hydrophobicity similar to that of the polymer matrix is an important factor in maintaining high stability and low transmission losses in POFs under high-temperature and high-humidity atmospheric conditions. However, although the stability and light transmission of POFs were significantly improved in these studies, POFs remain significantly affected by temperature and humidity. Furthermore, almost all of the work on the enhancement of the stability of POFs has focused mainly on their use in high-speed telecommunication and data communication networks^16^,^19^,^20^, and very few studies have been reported on the effects of high temperature and humidity conditions on the performance of FOEW sensors. Third, the temperature-dependent changes in the fiber’s physical properties—in particular, the surface morphology and deformation trajectory (fiber length, curvature, and diameter)—and their effects on the temperature independence and measurement consistency of the FOEW sensor have not been examined. More importantly, we have discovered that the measurement consistency and sensitivity of the POF FOEW sensors are not stable and vary with temperature in water. These observations have yet to be explained in the literature. Thus, an understanding of the temperature-dependent nature of POFs is required to facilitate the fabrication of high-quality FOEW sensors.

To determine the temperature independence of a U-shaped, polymer fiber-optic evanescent wave sensor and to exploit the advantages of polymer optical fibers, we investigated the possibility of the temperature-independent functioning of such a sensor. We examined the changes in the physical properties of the fiber-sensing region, including its surface morphology, deformation trajectory, refractive index, weight, and density, with varying temperature of the distilled water into which the sensing region was immersed. We also examined the spectral transmission and transmitted light intensity of fibers subjected to a heating-cooling treatment in the temperature range of −10 to 110 °C. In addition, we utilized the heat-treated fibers to realize a polymer fiber-optic evanescent wave sensor, and we evaluated the temperature independence and measurement consistency of our sensor using glucose solutions.

## Results

### Performance of POFs

**Figure 1:**
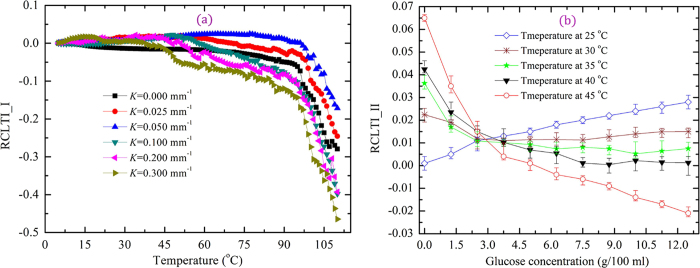
Performance of POF. RCTLI_I of (**a**) POFs with different curvatures as a function of temperature and (**b**) RCTLI_II as a function of glucose concentration.

To realize a high-performance POF FOEW sensor, we first investigated the relative change in the transmitted light intensity (RCTLI) of the D-shaped POF with different curvatures with varying distilled water temperature. The room temperature water was cooled to 5 °C and then was heated to 110 °C (the temperature glass transition of the PMMA is *T*_*g*_ = 105 °C)^21^,^22^. In Fig. 1a, the parameter RCTLI_I was defined as RCTLI_I = (*I*_T_ − *I*_1_)/*I*_1_, where *I*_1_ (45.2 nW) and *I*_T_ denote the transmitted light intensities of the POF at 5 °C and at temperature *T* (range of 5–110 °C), respectively. Thereafter, we examined the performance (consistency and sensibility) of the FOEW sensors with a curvature of 0.050 mm^−1^ based on the D-shaped POFs without heat-treated using glucose solutions as shown in Fig. 1b, where RCTLI_II = (*I*_T_ − *I*_a_)/*I*_a_, *I*_a_ (46.6 nW) and *I*_T_ denote the transmitted light intensities of the POF at 25 °C and at temperature 30, 35, 40, and 45 °C, respectively.

In the figure, note that the RCTLI_Is of the POFs with different curvatures are similarly affected by a change in the water temperature; the RCTLI_I initially slightly increases with an increase in temperature and subsequently decreases sharply above temperature of 98 °C. This result is in contrast to previous studies that have reported that the RCTLI_I, i.e., power loss, decreases with increasing temperature^18^. Consequently, to understand the increase in RCTLI_I in the initial phase, it is necessary to investigate the physical and optical properties of the U-shaped region as a function of the temperature of the distilled water.

Furthermore, in Fig. 1a, we first note that regarding the POFs with different curvatures, the light-transmission capacity of the POFs first increases and subsequently decreases with increasing curvature, and optimal light transmission is achieved with the POF with a curvature of 0.050 mm^−1^. Previous studies have hypothesized that the various phases of change in the RCTLI_Is can be attributed to the effect of skew rays on the bending losses (the bend radius of the fiber exceeds the critical angle necessary to confine the light to the core area; thus, there is leakage into the cladding), and the power loss increases with increasing curvature^23^,^24^. Consequently, this hypothesis indicates higher losses for POFs with larger curvatures but cannot completely explain our results because the studies in question were focused on optical communication instead of the functioning of optical sensors. In optical communications, the fiber cladding and even the coating of the fibers need not be removed, whereas in optical sensors, the coating and even sections of the fiber core are removed to sense changes in the sample and enhance the sensitivity of the sensors. The light transmission in fibers with a coating is different from that in thinned fibers, as described below. In the fiber-sensing region (U-shaped region), a large amount of light is lost at the interface between the fiber and the external environment via evanescent waves, scattering, refraction, and absorption. Furthermore, the power loss is also affected by the fiber angle of incidence, surface roughness, refractive index, geometry, and material composition^25^. In particular, the transmitted light intensity is controlled mainly by the angle of incidence for a given POF because the effective evanescent waves, reflection, refraction, and scattering intensity at the fiber–environment interface in the U-shaped region are controlled by the effective incidence angle^26^. The angle of incidence at the rough fiber surface first increases and subsequently decreases with increasing curvature, which leads to the power loss at the fiber–environment interface in a U-shaped region with a rough surface first decreases and then increases with increasing curvature. These trends can explain why the RCTLI_I first increases and then decreases with increasing fiber curvature. In light of the abovementioned results, we chose a POF with a curvature of 0.050 mm^−1^ for use in our subsequent experiments.

Figure 1b shows that the RCTLI_II of the sensor increases with increasing glucose concentration at 25 °C. However, the trends of the curves (RCTLI_IIs) vary markedly with increasing glucose concentration in the temperature range 25–45 °C, and the RCTLI_II decreases with increasing glucose concentration at 45 °C. The amount of change in the RCTLI_II at 45 °C is also larger than that at 25 °C, i.e., the sensor exhibits a higher sensitivity at 45 °C. As per references^25^,^27^, the increased RCTLI_II at 25 °C is explained as follows. First, the refractive index of the solution increases with increasing glucose concentration, thereby indicating that the difference in the refractive index between the fiber and glucose solution decreases. This trend further indicates that the random scattering and refraction of light at the fiber surface decrease. Second, in the glucose solution, light absorption is reduced in the spectral region of interest because of the low effective evanescent field intensity; hence, the transmitted intensity of the light is not affected by the evanescent field intensity according to evanescent wave theory. However, the above studies cannot explain the different trends of the curves (RCTLI_IIs) with increasing glucose concentration in the temperature range 25–45 °C or why the RCTL_II of the sensor at 45 °C is larger than that at 25 °C. Thus, to expain the findings presented in Fig. 1, it is necessary to investigate the effects of temperature and humidity on the physical and optical properties of the POFs.

### Physical and optical properties of POFs

**Figure 2:**
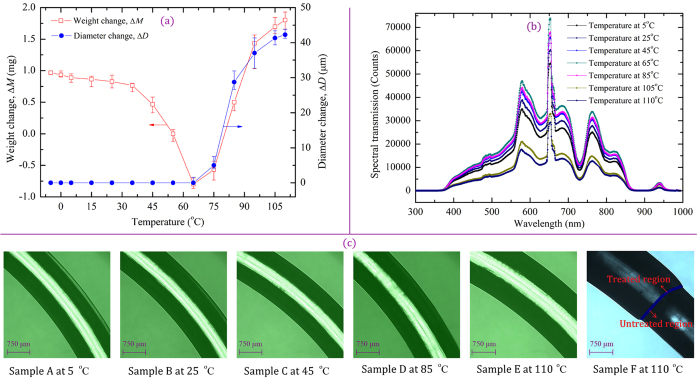
Weight, diameter, spectral transmission and micrograph images of the fibers. (**a**) Weight and diameter changes in fibers as a function of temperature, (**b**) spectral transmission of unclad fiber at different temperatures, and (**c**) optical micrograph images of the fiber diameters at a magnification of 100×.

To assess the temperature dependency of the light transmission of the POFs and the performance of the POF-based FOEW sensor, we studied the changes in the physical and optical properties of the D-shaped POFs with a curvature of 0.050 mm^−1^ with varying water temperature. In the experiments the U-shaped region with D-shaped POFs was immersed in the constant temperature bath and then was cooled to −5 °C; thereafter, the water was heated to 110 °C. We first verified the change in the weight and diameter of the POFs with increasing water temperature as shown in Fig. 2a. Subsequently, we examined the spectral transmission and optical micrographs of the POFs as shown in Fig. 2b,c. We defined the weight change as Δ*M* *=* *M*_T_ − *M*_1_, where *M*_1_ denotes the baseline weight at −5 °C (205.06 mg) and *M*_T_ denotes the post-treatment weight of the U-shaped region at *T* (−5 to 110  °C). We defined the diameter change as Δ*D* *=* *D*_T_ − *D*_1_, where *D*_1_ and *D*_T_ denote the lengths of the U-shaped region at −5 °C and *T* (−5 to 110  °C), respectively.

Figure 2a shows that the weight (weight change) of the U-shaped region first slowly decreases and subsequently rapidly decreases to a minimum at 65 °C. Thereafter, the weight significantly increases with increasing temperature. The initial decrease in weight is attributed to the readjustment of the uneven distribution of molecular weight and increase in the thermal decomposition of the residual monomers (vinyl-aromatic and ethylenically unsaturated monomers) in the polymers with increasing temperature^28^. The increased weight is from the fact that some amount of water molecules is absorbed by the POFs because the fiber core, i.e., PMMA, is in direct contact with the water, and the amount of absorbed water increases with increasing temperature and aging time^17^. The decomposition of the residual monomers can reduce the Rayleigh scattering losses in the fiber and increase the transmitted light intensity; however, when the water molecules are absorbed by the POF, much of the light is lost in the form of scattering because the absorbed water molecules are uniformly dispersed in the fiber core region and also as a result of O–H stretching vibration absorption^20^.

Figure 2b shows the spectral transmission of a U-shaped POF with a curvature of 0.050 mm^−1^. Clearly, the spectral transmission of the POF also first increases and subsequently rapidly decreases with increasing temperature. This result further confirmed that the experimental results in Fig. 1a; i.e., the transmitted light intensity (RCTLI_I) first increases and subsequently rapidly decreases with increasing water temperature.

Figure 2a,c show that the diameter (diameter change) of the U-shaped region is initially constant and subsequently rapidly increases with increasing temperature. A clear boundary (Fig. 2c, Sample F) between the U-shaped region (bare fiber) and the normal region (fiber with coating) was observed when the POF was heated to 110 °C in the water. The increased fiber diameter affects the core and cladding modes and consequently the performance of the FOEW sensor^24^,^29^. However, on the basis of the experimental result shown in Fig. 2 alone, it is very difficult to determine the effect of the fiber diameter on the light transmission and performance of the FOEW sensor; furthermore, the reason for the increased diameter (Fig. 2a) is unclear. Thus, further tests are required to elucidate the uncertainties involved.

**Figure 3:**
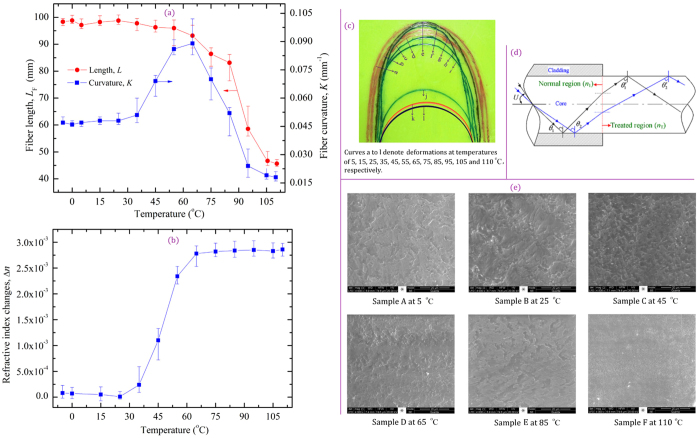
Thermal deformation, refractive and surface morphology of samples. (**a**) Fiber lengths and curvatures as a function of temperature, (**b**) refractive index change as a function of temperature, (**c**) the deformation (fiber length and curvature) of the U-shaped region with changing temperature, (**d**) the propagation path of light in the POF at 45 ^o^C, and (**e**) environmental scanning electron microscopy (ESEM) images (4.00 KX) of the fiber core.

In light of the above considerations, we examined the thermal deformation, i.e., the changes in fiber lengths and curvatures, of the U-shaped region of the POF samples as a function of water temperature. We further investigated the effect of varying the temperature on the refractive index and surface morphology of the fiber core. In the experiments, each sample was first cooled to −5 °C and then was heated to 110 °C using the constant temperature bath. We defined the refractive index change as Δ*n* *=* *n*_1_ − *n*_T_, where *n*_1_ and *n*_T_ denote the refractive indices of the U-shaped region at 25 °C a*n*d *T* (−5 to 110  °C), respectively. The experimental data and the obtained images are shown in Fig. 3.

Figure 3a shows that the length shrinkage of the U-shaped region isslow at first, and then decreases rapidly in the temperature range 65–95 °C and thereafter slows significantly when the temperature is above 105 °C, and the length decreases to approximately 46.5 mm at 110 °C. This behavior of the rapid decrease because of the change in the orientation of the polymer chain with increasing temperature; the POF exhibits large length shrinkage at high temperatures because of the fast shrinkage of the polymer chain^30^. However, when the temperature is increased to 105 °C, just below the glass transition temperature, the residual stresses of the PMMA decrease^31^, hence the shrinkage rate decreases. These facts explain why the fiber diameter increases with increasing temperature from 65 to 110 °C (Fig. 2a,c); the increased diameter is due to the length shrinkage.

In this regard, Sato *et al.*^30^ and Chen *et al.*^32^ have reported that the degree of shrinkage directly affects the light transmission in the fiber core, and fibers with large shrinkage exhibit a large increase in attenuation. Per the above mentioned studies, the effective transmitted light intensity of the POF at 25 °C is expected to be higher than that at 45 °C because the POF exhibits a smaller shrinkage at 25  °C. However, the effect of the length shrinkage on the light transmission can be neglected upon comparison with the effect of the weight loss on the light transmission in the temperature range −5–65 °C. Thus, for the D-shaped POF with a curvature of 0.050 mm^−1^, the transmitted light intensity increases with increasing temperature in the range of −5 to 65 °C, which explains the initial RCTLI_I increase in Fig. 1a.

Figure 3a shows that the curvatures of the U-shaped region exhibit four phases: a slow increase phase, a rapid increase phase, a rapid decrease phase, and a slow decrease phase. The deformation trajectory of the length and curvature of the U-shaped region was also recorded by tracing the profile of the heat-treated POF at different temperatures (Fig. 3c). The curvature increase arises because the fiber strength decreases with increasing temperature. However, when the temperature is above 65 °C, the POF rapidly deforms and shortens, thereby leading to POF aging and mechanical property (strength) increase; consequently, the curvatures increase. The curvatures of the U-shaped region slowly decrease, because the POF undergoes the glass transition and the spontaneous reforming of the polymer bonds^31^,^33^. The sensitivity of FOEW sensors increases with increasing curvature, which indicates that the sensitivity of the POF FOEW sensor also first increases and then decreases with increasing temperature^34^. Thus, the sensor exhibits a higher sensitivity at 45 °C than at 25 °C because of the higher transmitted light intensity and larger curvature^28^. This observation also validates the experimental results shown in Fig. 1b—i.e., the RCTLI_II (sensitivity) of the sensor at 45 °C is larger than that at 25 °C; however, the different trends of the curves (RCTLI_IIs) with increasing glucose concentration in the temperature range 25–45 °C cannot be explained. Thus, the experiments of Fig. 3b,e are necessary to determine the effect of the water temperature on the performance of POF-based FOEW sensors.

Figure 3b shows that the refractive index change (Δ*n*) remains nearly unchanged with increasing temperature in the range of −5 to 25 °C and then rapidly increases, thereafter slightly increases when the temperature is above 60 °C. The unchanged refractive index, or Δ*n*, results from the POF weight and deformations, including the diameter, length, and curvature, being nearly unchanged in the temperature range −5–25 °C. The sharply increased Δ*n*, i.e. decreased refractive index, in the temperature range of 25 to 60 °C can be explained as follows. Although the refractive index of PMMA slightly increases with increasing absorbed moisture^35^, the refractive index mainly depends on the negative thermo-optic coefficient of PMMA^36^. However, Δ*n* shows only a slight increase, this behavior can be attributed to the balance among the moisture sorption, deformation (diameter, length and curvature), and negative thermo-optic coefficient^35^,^36^,^37^. Furthermore, Fig. 3e shows that the surface roughness decreases and that a dense structure gradually emerges with increasing temperature in the range from 25 to 110 °C. This behavior can be attributed to the thermal decomposition of the residual monomers and thermal shrinkage of the PMMA material in the POF core^33^.

We next analyzed the effect of the refractive index change on the path of the transmitted light (in the temperature range 25–45 °C). The increase in Δ*n* changes the propagation path of light as shown in Fig. 3d. We note that light is refracted at the interface between the normal region with refractive index *n*_1_ and the treated region with refractive index *n*_T_ (*n*_T_ < *n*_1_), thereby leading to an decrease in the angle of incidence (*θ*), i.e., the angle decreases from *θ* to *θ‘*. Although the decrease in the incidence angle can increase the refraction at the interface between the fiber and the external environment in the U-shaped region, the decrease in the surface roughness can reduce scattering losses and refraction at the interface, thereby leading to the fact that the amount of totally internally reflected light increases with decreasing surface roughness^28^; hence, the effective evanescent wave intensity increases. The increased effective evanescent wave intensity can enhance the attenuation of the evanescent waves, and the attenuation increases with increasing glucose concentration. Furthermore, the RCTLI_I increases with an increase in temperature range of 25–45 °C (Figs 1a and 2b). These can be used to explain the different trends of the curves (RCTLI_IIs) with increasing glucose concentration in the temperature range 25–45 °C.

The above analysis clearly shows the effect of the water temperature on the physical and optical properties of the POF FOEW sensors. However, the focus of this study was also to ensure the stability of the physical and optical properties of the POF and to realize a high-performance sensor. This aspect of the study is presented in the following section.

### High-performance U-shaped FOEW sensor using heating-cooling-treated POFs

**Figure 4:**
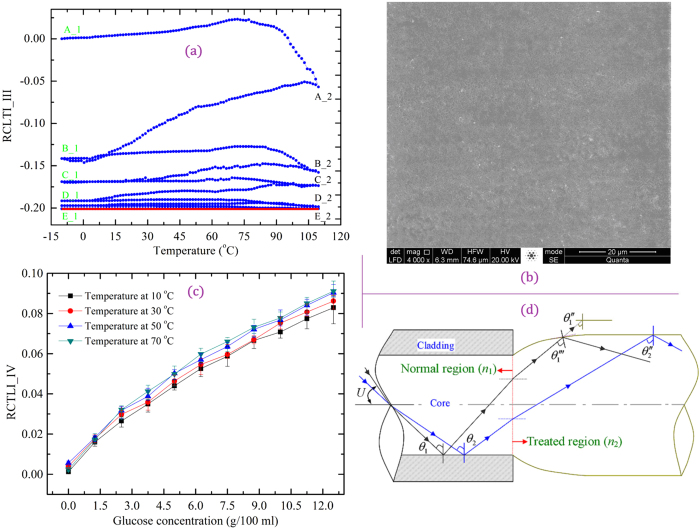
Change trajectory of light, surface morphology and temperature independence of the POF sensor. (**a**) RCTLI_III as a function of temperature (curves A_1, B_1, C_1, D_1, and E_1 represent RCTLI_III values over the temperature range of −10 to 110 °C, and curves A_2, B_2, C_2, D_2, and E_2 represent RCTLI_III values over the range of 110 to −10 °C), (b) ESEM image (4.00 kX) of U-shaped region subjected to heating-cooling treatment, (**c**) RCTLI_IV as a function of glucose concentration, and (**D**) the propagation path of light in the heating-cooling-treated POF.

To realize a temperature-independent measuring consistency and high-sensitivity POF sensor, we fabricated a U-shaped FOEW sensor using the D-shaped POF with a curvature of 0.050 mm^−1^. The U-shaped region of the sensor was subject to numerous rounds of heating-cooling treatment in the temperature range of −10 to110 °C by alternately heating and cooling using the constant temperature bath. We studied the change trajectory of the RCTLI_III with changing water temperature; we also observed the heat-treated surface morphology and the temperature independence of the POF sensor. The experimental results are shown in Fig. 4. In Fig. 4a, the parameter RCTLI_III was defined as RCTLI_III = (*I*_T_ *– I*_c_)/*I*_c_, where *I*_c_ (32.7 nW) and *I*_T_ denote the transmitted light intensitiesof the POF at −10 °C and at temperature *T* (range of −10–110 °C), respectively; in Fig. 4c, the parameter RCTLI_IV was defined as RCTLI_IV = (*I*_d_ *– I*_T_)/*I*_d_, where *I*_d_ (26.9 nW) and *I*_T_ denote the transmitted light intensities of the POF at 10 °C and at temperature *T* (range of 10–70 °C).

Figure 4a clearly shows the change in trajectory of the RCTLI_III curve with changing water temperature, the RCTLI_III exhibits a minimum, and it maintains a constant when the U-shaped region is subjected to five rounds of heating-cooling treatments. This RCTLI_III minimum can be attributed to the increase in the fiber deformation (the diameter, length, and curvature were transformed to approximately 1054 μm, 44.8 mm, and 0.014 mm^−1^, respectively) and the amount of absorbed water in the fiber core (the fiber weight increased by approximately 1.83 mg) after five heating-cooling treatments. The constant level of the RCTLI_III is from the fact that the glass transition of the PMMA and the spontaneous reforming of the polymer bonds have been completed^31^,^33^. Meanwhile, the low transmitted light intensity implies an increased light attenuation by Rayleigh scattering and by the absorption of water molecules in the fiber. On the other hand, the Mie scattering attenuation and refraction at the fiber–environment interface decreases when the U-shaped region is subjected to heating-cooling treatment because the surface roughness decreases (the scattering attenuation and refraction increase with increasing roughness^25^,^38^) and the PMMA experienced glass transition, as shown in Fig. 4b. Thus, the sensitivity of the heat-treated sensor depends mainly on the evanescent wave intensity at the fiber–environment interface.

Figure 4c shows that the RCTLI_IV of the heating-cooling-treated U-shaped POF sensor exhibits the same trend in the temperature range of 10 to 70 °C with increasing glucose concentration (70 °C is the upper limit of the operating temperature of the POF). This is because the heating-cooling-treated U-shaped (sensing) region has a stable geometry, weight, refractive index, and light transmission, and these parameters are not affected by temperature variations over the range of 10 to 70 °C. The deviation of 9.7% between the two curves at temperatures of 10 and 70 °C can be attributed to the change in the spacing of water and glucose molecules with increasing temperature^39^.

We now discuss the increased RCTLI_IV observed in the sensor with increasing glucose concentration. First, the increases in the refractive index change (Δ*n*) and diameter (Fig. 2c, Sample F) change the propagation path of light (Fig. 4d). Although the angle of incidence *θ*_1_ will decrease to *θ*_1_′′ because of the decrease in the refractive index, it will increase to *θ*_1_′′′ and have *θ*_1_′′′ > *θ*_1_ because of the increase in the fiber diameter (Fig. 4d). The increase in the incidence angle leads to increased total internal reflection at the fiber–environment interface; thus, the refraction attenuation decreases. Second, the decrease in the surface roughness reduces the Mie scattering and refraction losses. Thus, the sensitivity of the heating-cooling-treated POF sensor is dependent on the attenuation of the evanescent field by the sample medium. Furthermore, a comparison of Fig. 4c with Fig. 1b, indicates that the heating-cooling-treated U-shaped (sensing) region has high sensitivity, which can be attributed to the optimal surface roughness of the sensing region, the reduction in refraction and scattering losses, and enhancement of the evanescent wave intensity at the fiber-medium interface.

## Discussion

While the numerous advantages of polymer optical fibers (low cost, large-diameter core, etc.) have been exploited in sensors and telecommunications, such fibers still experience a critical problem of the temperature dependency. Hence, we explored the temperature-independent operation of a POF FOEW sensor immersed in distilled water and observed the resultant phenomena. We summarize our findings as follows.

The RCTLI_I of the POF initially increased with increasing temperature because the uneven distribution of molecular weight was evenly redistributed and because the thermal decomposition of the residual monomers in polymers increased, leading to a reduction in losses due to Rayleigh scattering.

The light-transmission capacity of the POFs first increased and then decreased with increasing curvature for a given temperature; this pattern can be attributed to the change in the angle of incidence at the rough fiber surface with varying curvature and roughness, consequently changing the core and cladding modes (light-transmission modes) in the U-shaped region.

The RCTLI_II of the POF-based U-shaped sensor increased with increasing glucose concentration at 25 °C, primarily because the difference in the refractive index between the U-shaped (sensing) region with large roughness and the external environment decreased with increasing glucose concentration and the reduced evanescent wave absorption. Thus, the random scattering and refraction of light at the fiber surface decreased with increasing glucose concentration.

The different trends of curves (RCTLI_IIs) with increasing glucose concentration in the temperature range 25–45 °C, and the RCTLI_II of the POF U-shaped sensor significantly decreased with increasing glucose concentration at 45 °C. These facts can be explained as follows. The first was the increased transmitted light intensity of the POFs in the temperature range 25–45 °C. The second was the decrease in the surface roughness, thereby leading to decreased scattering and refraction at the fiber–environment interface and increased evanescent wave intensity; this leading to the transmitted light intensity of the POFs is significantly affected by the attenuation of evanescent waves; in addition, the attenuation increases with increasing glucose concentration. Furthermore, the high sensitivity (RCTLI_II) was due to the high transmitted light intensity (resulting from the weight loss caused by the readjustment of the molecular weight and the thermal decomposition of the residual monomers) and the sensing region’s larger curvature at 45 °C compared with that at 25 °C.

In addition, we fabricated a temperature-independent U-shaped POF FOEW sensor by subjecting the sensing region to heating-cooling treatment. The fabricated POF-based U-shaped sensor showed a significant temperature independence, high sensitivity and high degree of consistency in measurements of glucose solution concentrations. The temperature independence and measuring consistency of the sensor are attributable to the fact that the repeated heating-cooling of POFs led to the glass transition of the PMMA, the high stability of their physical and optical properties. The RCTLI_IV increase observed in the sensor with increasing glucose concentration can be explained as follows. First, the decrease in the refractive index and the increase in the diameter changed the propagation path of light and increased the total internal reflection at the fiber–environment interface; Second, the increase in smoothness reduced the Mie scattering losses, and the scattering losses from Mie and Rayleigh scattering, particularly Rayleigh scattering, which increased the evanescent wave intensity at the fiber–environment interface. The higher sensitivity of the heating-cooling-treated POF FOEW sensor (RCTLI_IV) than that of untreated POF-based FOEW sensor (RCTLI_II) was resulted from attaining optimal surface roughness of the sensing region, reduction in refraction and scattering losses and high attenuation of the evanescent field.

In summary, our investigation of the temperature dependence of POF-based FOEWs and our fabricated temperature-independent sensor can be used to significantly improve the performance of POF-based sensors and extend their application in optical sensing. We believe that our findings can significantly contribute to the development of POF-based FOEW sensors, telecommunication and data networks.

## Materials and methods

### Materials

We used plastic optical fibers [Jiangxi Daishing (China) POF Co., Ltd., Jiangxi, China; fiber diameters and cladding diameters of 2200 ± 50 and 1500 ± 5 μm] with a core material made of PMMA, fiber cladding material composed of fluorinated polymer, numerical aperture of 0.5, core refractive index of 1.49, and operating temperature range of −50 to 70 °C, respectively. In this work, the studied POFs region was prepared as follows. First, the coatings of the fibers were removed over a length of 100 mm using an fiber optic stripping tool (84–870, Stanley, USA), and subsequently, the D-shaped (sensing region) fiber was prepared by polishing the uncoated region held by a V grooved block; the uncoated region of the fiber together with the V-grooved block was polished by precise machining (YM-36XL, Shenzhen Dajing Grinding Technology Co., Ltd, Shenzhen, China), and the polishing depth of the uncoated fiber was 500 ±5 μm. Second, the D-shaped region of the POFs was used to create a U-shaped FOEW sensor, herein, the normal (non-sensing) region of the POFs was affixed to a U-shaped steel mold with a small thermal deformation (the normal regions with the POF fiber coating were affixed to the steel mold such that the D-shaped POF region could deform with varying temperature). The U-shaped region with D-shaped POFs was used as the object of study in this work.

### Experimental conditions and analytical methods

We investigated the changes in the surface morphology, deformation trajectory, refractive index, and weight of the D-shaped fiber region with varying temperature of distilled water. Regarding the heating-cooling treatment, the temperature of the distilled water was controlled using a high & low constant temperature bath with operating temperature range of −40 to 200 °C, temperature fluctuation of ±0.1 °C, liquid tank of 5 L, heating power of 1500 W, and refrigerating capacity of 900 W at temperature 0 °C, respectively (BILON-CXWD-505, Tianjin Bilon Lab Equipment Co., Ltd. Tianjin, China); in this work, the heating (cooling) rate of the water was set at 1 °C/min.

The moisture on the surface of the heating-cooling-treated fibers was dried at 25 °C using argon gas; the heating-cooling-treated fibers’ surface morphology, diameter, length, curvature, refractive index, and weight were analyzed at room temperature (about 25 °C). The surface morphology of the heating-cooling-treated fibers (D-shaped fiber region) was examined using an environmental scanning electron microscope (ESEM, Quanta 200, FEI, USA) operating at 25 °C. An optical microscope system (IX81, Olympus, Japan) with a resolution of ±1 μm was used to measure the diameter of the fibers. The D-shaped fiber length and curvature were monitored using a digital micrometer with a resolution of 1 μm. The refractive index of the fibers (D-shaped fiber region) was measured by a refractometer with a resolution of ±0.0002 (NAR-1T solid, ATAGO, Japan). The weight of the fibers (D-shaped fiber region) was determined using a microbalance with a resolution of 1 μg (XP56, Mettler Toledo). The weight loss and gain of the fibers were determined by comparing the weight of the heating-cooling-treated fibers with their baseline weight. To measure the spectral quality and transmitted light intensity of the fibers, both ends of the POFs were polished and connected to standard SMA 905 connectors. The light from a deuterium tungsten halogen light source (25-W deuterium, 20-W tungsten halogen) emitting light in the wavelength range of 190–2000 nm (DH-2000, Oceanoptics, USA) was transmitted through the fibers. The transmitted spectrum of the fibers was measured with an optical spectrometer (QE65000, Oceanoptics, USA) with a spectral resolution of 0.14–7.7 nm over a wavelength range of 200–1150 nm. The transmitted-light intensity was measured using a power meter (UV 0.2, Newport Corporation, USA, obtained from the NBeT Group Corp., China) with a power range of 100 pW to 0.2 W and maximum uncertainty of 4% over a wavelength range of 200–1100 nm. Each experiment was repeated more than five times.

## Additional Information

**How to cite this article**: Zhong, N. *et al.* Temperature-independent polymer optical fiber evanescent wave sensor. *Sci. Rep.* **5**, 11508; doi: 10.1038/srep11508 (2015).
